# Bloodstream infection and ventilator-associated pneumonia in patients with coronavirus disease 2019 (COVID-19) supported by extracorporeal membrane oxygenation

**DOI:** 10.1017/ice.2022.290

**Published:** 2023-09

**Authors:** Charlie Tan, Susy S. Hota, Eddy Fan, Krista Marquis, Elisa Vicencio, Alon Vaisman

**Affiliations:** 1 Division of Infectious Diseases, University of Toronto, Toronto, Ontario, Canada; 2 Infection Prevention and Control, University Health Network, Toronto, Ontario, Canada; 3 Interdepartmental Division of Critical Care, University of Toronto, Toronto, Ontario, Canada

## Abstract

**Objective:**

Extracorporeal membrane oxygenation (ECMO) has been widely used in the care of patients with respiratory failure from coronavirus disease 2019 (COVID-19). We characterized bloodstream infections (BSIs) and ventilator-associated pneumonias (VAPs) in COVID-19 patients supported with ECMO, and we investigated their impact on patient outcomes.

**Design:**

Retrospective cohort study from March 1, 2020, to June 30, 2021.

**Setting:**

Academic tertiary-care referral center.

**Patients:**

Consecutive adult patients admitted for COVID-19 who received ECMO.

**Methods:**

We identified BSIs and VAPs and described their epidemiology and microbiology. Cumulative antimicrobial use and the specific management of BSIs were determined. Multivariate time-dependent Cox proportional hazards models were constructed to evaluate the impact of BSIs and VAPs on mortality, controlling for age, receipt of COVID-19–specific therapeutics, and new renal replacement therapy.

**Results:**

We identified 136 patients who received ECMO for COVID-19 pneumonia during the study period. BSIs and VAPs occurred in 81 patients (59.6%) and 93 patients (68.4%), respectively. The incidence of BSIs was 29.5 per 1,000 ECMO days and increased with duration of ECMO cannulation. Enterococci, Enterobacterales, and *Staphylococcus aureus* were the most common causes of BSIs, whereas *S. aureus*, *Klebsiella* species, and *Pseudomonas aeruginosa* comprised the majority of VAPs. Mean antibiotic use comprised 1,031 days of therapy per 1,000 ECMO days (SD, 496). We did not detect an association between BSIs or VAPs and mortality.

**Conclusions:**

BSIs and VAPs are common in COVID-19 ECMO-supported patients. Efforts to optimize their diagnosis, prevention, and management should be prioritized.

Extracorporeal membrane oxygenation (ECMO) is a form of extracorporeal life support for respiratory or cardiac failure refractory to conventional therapies. Venovenous ECMO has been widely employed during the coronavirus disease 2019 (COVID-19) pandemic for patients with severe COVID-19–related respiratory failure.^1^ Despite early experience from Wuhan, China, suggesting that ECMO may be ineffective for this indication,^2^ subsequent studies have shown mortality rates similar to those in non–COVID-19 ECMO-supported patients.^1,3–7^ ECMO is now included in guidelines for the management of COVID-19.^8,9^

Healthcare-associated infections (HAIs) are common in patients receiving ECMO. Incidence estimates have been variable, ranging from 11.9 to 75.5 infections per 1,000 ECMO days in adult patients.^10^ Patients supported with ECMO are at high risk of HAIs, especially bloodstream infections (BSIs) and ventilator-associated pneumonias (VAPs), for several reasons. ECMO may necessitate prolonged vascular cannulation. Recipients also typically have additional indwelling devices (eg, central venous catheters, hemodialysis catheters) and require extended mechanical ventilation. HAIs in ECMO recipients have been associated with adverse outcomes, including increased ECMO duration and higher mortality.^11,12^

However, HAIs in ECMO-supported patients remain poorly characterized. Few studies have evaluated HAIs in adult patients, and they differ widely in their described epidemiology and microbiology.^13–16^ Registry studies have attempted to compile data across multiple centers, but they do not provide details on the types of infections encountered.^11,12^ Literature on HAIs in COVID-19 patients on ECMO is particularly scarce.^17,18^ Furthermore, the management of such infections has not been well studied. Treatment, especially of BSIs, is complicated; standard principles are challenging to apply due to long-term central catheterization that cannot be readily removed, increasing risk of persistence or relapse. Understanding the types of infections COVID-19 patients supported with ECMO develop, the culprit pathogens, and their optimal management are critical to better therapeutic and preventative measures. Therefore, we aimed to describe the epidemiology, microbiology, and treatment of BSIs and VAPs in COVID-19 ECMO-supported patients, and we evaluated their impact on patient outcomes.

## Methods

### Study design and data sources

We conducted a retrospective cohort study of COVID-19 patients admitted to Toronto General Hospital between March 1, 2020, and June 30, 2021, who received ECMO. Our hospital is the largest adult ECMO program in Canada and serves as a regional referral center. The study period spanned the first to third waves of the COVID-19 pandemic in Ontario. Patients were identified using data collected by the infection prevention and control department, which keeps a record of all ECMO recipients. We included all consecutive patients aged ≥18 years with positive severe acute respiratory coronavirus virus 2 (SARS-CoV-2) polymerase chain reaction (PCR) from an upper or lower respiratory tract specimen and ECMO cannulation for ≥24 hours. This study was approved by the research ethics board of the University Health Network. Patient consent was waived due to anonymization of data and the retrospective design.

### Infection prevention procedures for patients on ECMO

ECMO cannulation at our center is performed in operating rooms following a surgical protocol. All patients on ECMO receive routine 2% chlorhexidine bathing. Dressings over ECMO cannula are changed using sterile gloves every 7 days or when visibly soiled. Standard bundles to reduce central-line–associated BSIs (CLABSIs) and VAPs are used for all patients in our intensive care unit (ICU) who have central venous catheters and/or who receive mechanical ventilation. Standard antimicrobial prophylaxis is not offered to patients receiving ECMO at our center.

### Definitions of HAIs

BSIs and VAPs that occurred following ECMO cannulation and up to 48 hours after decannulation were included. The National Healthcare Safety Network (NHSN) laboratory-confirmed BSI criteria^19^ were used. Pathogens on the NHSN common commensals list were included if they were positive in ≥2 blood cultures collected separately and the patient had systemic features of infection. Persistence was defined as consecutive blood cultures positive for the same organism for ≥5 days. Relapse was defined as culturing a previously cleared organism, collected ≥48 hours after a blood culture demonstrating clearance.

VAPs are challenging to define in patients with COVID-19 pneumonia on ECMO.^20^ NHSN criteria for VAP, based on radiographic changes, fever, leukocytosis or leukopenia, new respiratory symptoms, and worsening ventilatory status^21^ cannot be accurately applied to patients with COVID-19 who present with overlapping clinical manifestations. Patients with severe COVID-19 on ECMO have extensive baseline radiographic infiltrates, which limits the assessment of superimposed pneumonia. Quantitative or semiquantitative cultures of lower respiratory specimens (endotracheal aspirate or bronchoalveolar lavage) support a diagnosis of VAP, but patients on long-term mechanical ventilation often have asymptomatic airway colonization without true infection. In this study, VAP was defined as a positive semiquantitative endotracheal aspirate culture or quantitative bronchoalveolar lavage culture of an NHSN-eligible organism that was treated with antimicrobials. We had concerns about overestimating VAP incidence with this definition because patients were often treated with multiple antimicrobial courses for respiratory cultures that were repeatedly positive. Therefore, only the proportion of patients who developed a VAP was calculated.

### Data collection

The data abstracted for each patient included age, sex, comorbidities, dates of hospital admission and discharge, dates of ICU admission and discharge, date of positive SARS-CoV-2 PCR, dates of intubation and extubation, ECMO configuration (venovenous, venoarterial, other), dates of ECMO cannulation and decannulation, receipt of COVID-19–specific therapeutics (dexamethasone, remdesivir, interleukin-6 inhibitors), lung transplantation, new renal replacement therapy, *Clostridioides difficile* infection (defined by positive toxin PCR), and disposition at discharge. Comorbidities included hypertension, dyslipidemia, diabetes mellitus, coronary artery disease, congestive heart failure, cerebrovascular disease, chronic pulmonary disease (including asthma, chronic obstructive pulmonary disease, interstitial lung disease), chronic kidney disease, cirrhosis, malignancy (active or treatment within past 6 months), and immunocompromise (including HIV infection with CD4 count <200 cells/mm^3^, solid-organ or bone marrow transplant, immunosuppressing medications). Dispositions at discharge were death, transfer to other hospital, discharge to a rehabilitation facility, or discharge home. The dates and organisms of all positive blood cultures, respiratory cultures, and pleural fluid cultures were abstracted. We also collected the regimens and durations of all antibiotics and antifungals received.

### Statistical analyses

The study cohort was described by patient and hospitalization characteristics. Patients with BSIs were compared against patients without BSIs. Continuous variables were reported as means with standard deviations (SDs) or as medians with interquartile ranges (IQRs), depending on their distribution. Categorical variables were reported as frequencies and percentages.

We calculated the proportion of patients who developed BSIs and VAPs. We also determined the incidence of BSIs, calculated as the number of BSIs per 1,000 ECMO days. A Kaplan-Meier analysis was performed to determine the probability of being BSI free for the duration of ECMO support. The organisms identified on blood and respiratory cultures were compiled to describe the microbiology of BSIs and VAPs, respectively.

To evaluate antimicrobial use among COVID-19 ECMO-supported patients, we aggregated the antibiotics and antifungals administered to each patient while receiving ECMO. Antimicrobial use was measured in days of therapy (DOT) per 1,000 ECMO days. The duration of appropriate antimicrobial therapy for each BSI isolate was also determined. An appropriate antimicrobial was one to which the BSI organism was susceptible on drug susceptibility testing. Duration was calculated from the day of antimicrobial initiation to the day of discontinuation, either as a clinical decision or due to patient death or transfer to another hospital. Treatment was classified by whether therapy was stopped before or continued until ECMO decannulation. We excluded BSI isolates treated with <7 days of antimicrobials due to death or transfer.

We constructed 2 multivariate time-dependent Cox proportional hazards models to investigate the association (1) between BSI and mortality and (2) between VAP and mortality. The variables included in both models were BSI or VAP, patient age, receipt of dexamethasone, receipt of tocilizumab, and new renal replacement therapy, selected a priori based on expected clinical significance. Time at risk was defined as the date of ECMO cannulation to date of discharge, transfer, or death. To account for immortal time bias, BSI and VAP were treated as time-dependent variables. All statistical analyses were performed with R version 4.1.0 software (R Foundation for Statistical Computing, Vienna, Austria).

## Results

We identified 136 patients during the study period who received ECMO for COVID-19–related respiratory failure. Baseline patient characteristics are shown in Table 1. The median age was 49 years (IQR, 42–54) and 106 (77.9%) were men. Only 4 (2.9%) were admitted directly; the remainder were transferred from a regional center for ECMO. Also, 70 patients (51.5%) had no comorbidities, but 35 (25.7%) had hypertension, 25 (18.4%) had diabetes mellitus, and 21 (15.4%) had dyslipidemia. Dexamethasone was administered to 112 patients (82.4%); interleukin-6 inhibitors and remdesivir were administered to 62 patients (45.6%) and 18 patients (13.2%), respectively.

**Table 1.** Clinical Characteristics of Extracorporeal Membrane Oxygenation-Supported Patients, With and Without Bloodstream Infection

Variable	All Patients (n = 136), No. (%)^a^	Bloodstream Infection (n = 81), No. (%)^a^	No Bloodstream Infection (n = 55), No. (%)^a^
Age, median y (IQR)	49 (42–54)	50 (46–55)	48 (39–54)
Sex, male	106 (76.8)	65 (80.2)	41 (74.5)
**Pandemic wave**			
1	33 (24.3)	14 (17.3)	19 (34.5)
2	39 (28.7)	25 (30.9)	14 (25.5)
3	64 (47.1)	42 (51.9)	22 (40.0)
**Comorbidities**			
Hypertension	35 (25.7)	19 (23.5)	16 (29.1)
Dyslipidemia	21 (15.4)	14 (17.3)	7 (12.7)
Diabetes mellitus	25 (18.4)	14 (17.3)	11 (20.0)
Coronary artery disease	2 (1.5)	0 (0)	2 (3.6)
Congestive heart failure	1 (0.7)	0 (0)	1 (1.8)
Cerebrovascular disease	0 (0)	0 (0)	0 (0)
Chronic lung disease	17 (12.5)	12 (14.8)	5 (0.9)
Chronic kidney disease	2 (1.5)	1 (1.2)	1 (1.8)
Cancer	1 (0.7)	1 (1.2)	0 (0)
Immunocompromise	3 (2.2)	2 (2.5)	1 (1.8)
**COVID-19 therapies**			
Dexamethasone	112 (82.4)	72 (88.9)	40 (72.7)
Remdesivir	18 (13.2)	11 (13.6)	7 (12.7)
Interleukin-6 inhibitor	62 (45.6)	38 (46.9)	24 (43.6)
**Type of ECMO**			
Venovenous	132 (97.1)	79 (97.5)	53 (96.4)
Venoarterial	2 (1.5)	0 (0)	2 (3.6)
Both	2 (1.5)	2 (2.5)	0 (0)
Time from initial hospital admission to ECMO, median d (IQR)	10 (6–14)	10 (6–14)	10 (5–12)
Time from mechanical ventilation to ECMO, median d (IQR)	5 (2–8)	4 (1–7)	5 (2–8)
Duration of admission at study center, median d (IQR)	29 (18–52)	40 (23–66)	19 (12–31)
Duration of mechanical ventilation, median d (IQR)	32 (19–53)	45 (26–62)	22 (13–32)
Duration of ECMO, median d (IQR)	24 (12–48)	32 (18–55)	13 (7–24)
New renal replacement therapy	56 (41.2)	39 (48.1)	17 (30.9)
*Clostridioides difficile* infection	2 (1.5)	2 (2.5)	0 (0)
Lung transplantation	1 (0.7)	1 (1.2)	0 (0)
**Patient outcome**			
Transfer to other hospital	49 (36.0)	29 (35.8)	20 (36.4)
Discharge to rehabilitation facility	6 (4.4)	4 (4.9)	2 (3.6)
Discharge home	6 (4.4)	2 (2.5)	4 (7.3)
Death	75 (55.1)	46 (56.8)	29 (52.7)

Note. COVID-19, coronavirus disease 2019; ECMO, extracorporeal membrane oxygenation; IQR, interquartile range.

a Units unless otherwise specified.

Among the 136 patients in the study cohort, 132 (97.6%) received venovenous ECMO. The median time from first hospital admission (including days hospitalized at transferring institution) to ECMO cannulation was 10 days (IQR, 6–14). The median time from positive SARS-CoV-2 PCR was 12 days (IQR, 8–18). The median time from intubation was 5 days (IQR, 2–8). Patients remained cannulated for a median of 24 days (IQR, 12–48) and received mechanical ventilation for a median of 32 days (IQR, 19–53). Renal replacement therapy was initiated in 56 patients (41.2%). Also, 2 patients developed *C. difficile* colitis and 75 patients (55.1%) died in hospital. Of these 75 patients, 69 (92.0%) were still on ECMO at time of death.

**Fig. 1.**
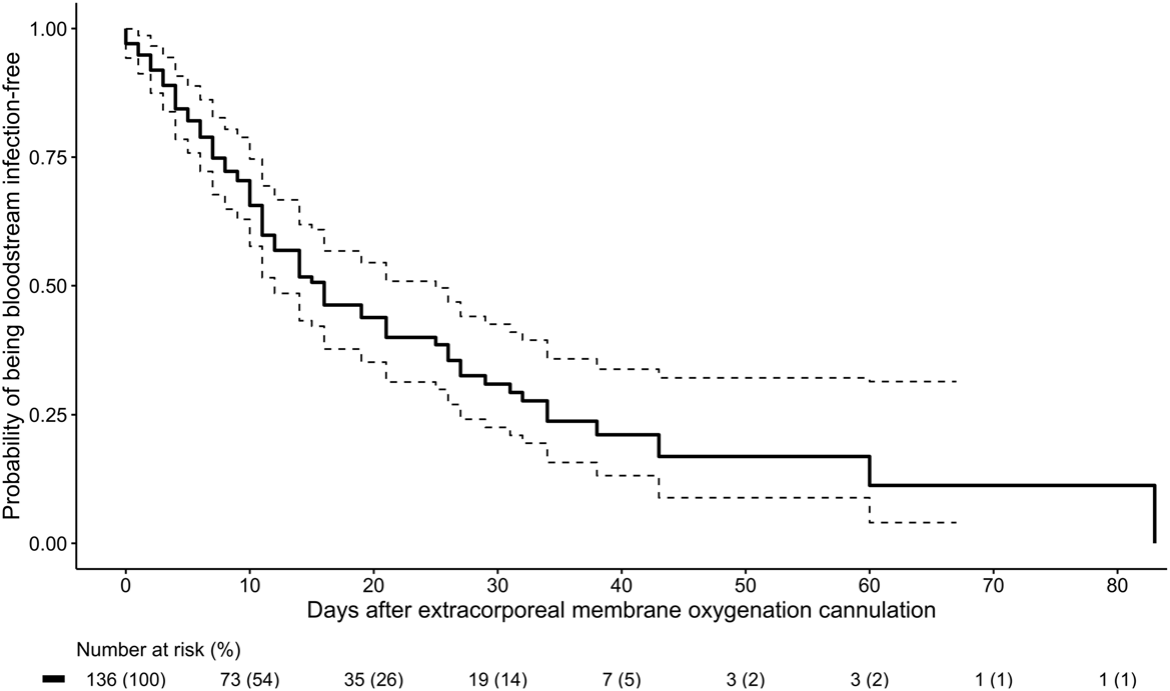
Kaplan-Meier curve of probability of being free of bloodstream infection (solid line) by days of extracorporeal membrane oxygenation cannulation, with 95% confidence interval (dotted lines).

In this cohort of 136 ECMO recipients, 81 (59.6%) had BSIs. Among them, 126 were discrete BSIs with 4,271 total days of ECMO cannulation, for an incidence of 29.5 BSIs per 1,000 ECMO days. The wave-specific incidences in the first, second, and third waves of the pandemic were 30.6, 32.6, and 27.5 BSIs per 1,000 ECMO days, respectively. Of the 81 patients with BSIs, 49 (60.5%) had 1 distinct episode; 22 (27.2%) had 2 episodes; and 10 (9.9%) had ≥3 episodes. The Kaplan-Meier curve for probability of being BSI-free is shown in Figure 1. The likelihood of being BSI-free declined over time: 74.8% on day 7 of ECMO cannulation (95% confidence interval [CI], 67.7%–82.7%), 51.7% on day 14 (95% CI, 43.2%–61.9%), and 39.9% on day 21 (95% CI, 31.4%–50.9%). The microbiology of the 142 BSI isolates are listed in Table 2. Of these 142 isolates, 34 (23.9%) were enterococci and 30 (21.1%) were *Staphylococcus aureus*. Gram-negative bacilli comprised 48 (33.8%) of all isolates; 33 (23.2%) were Enterobacterales and 10 (7.0%) were glucose nonfermenters. Also, 15 (10.6%) were *Candida*. BSIs were persistent in 23 (18.3%) of 126 cases, and relapse occurred in 28 patients (22.2%). BSIs were not associated with mortality (unadjusted hazard ratio [HR], 1.12; 95% CI, 0.61–2.02), including after adjustment for covariates (adjusted HR, 1.14; 95% CI, 0.63–2.06). The multivariate model is shown in Table 3A.

**Table 2.** Microbiology of Bloodstream Infection Isolates

Microorganism	Total (n = 142), No. (%)
**Gram positive**	**79 (55.6)**
*Enterococcus faecalis*	28 (19.7)
Methicillin-susceptible *Staphylococcus aureus*	28 (19.7)
Coagulase-negative staphylococci	6 (4.2)
*Enterococcus faecium*	6 (4.2)
Methicillin-resistant *Staphylococcus aureus*	2 (1.4)
Other gram positive organisms	9 (6.3)
**Gram negative**	**48 (33.8)**
*Klebsiella* spp	19 (13.4)
*Pseudomonas aeruginosa*	7 (4.9)
*Escherichia coli*	5 (3.5)
*Enterobacter cloacae*	3 (2.1)
*Citrobacter koseri*	3 (2.1)
Other gram-negative organisms	11 (7.7)
***Candida***	**15 (10.6)**
*Candida parapsilosis*	9 (6.3)
*Candida albicans*	3 (2.1)
Other *Candida* spp	3 (2.1)

**Table 3.** Multivariate Time-Dependent Cox Proportional Hazards Models for Association Between (A) Bloodstream Infection and Mortality, and (B) Ventilator-Associated Pneumonia and Mortality

Variable	Hazard Ratio (95% CI)
**(A)**	
Bloodstream infection	1.14 (0.63–2.06)
Age	1.02 (0.99–1.05)
Administration of dexamethasone	0.69 (0.31–1.53)
Administration of tocilizumab	1.29 (0.74–2.24)
New renal replacement therapy	1.03 (0.62–1.70)
**(B)**	
Ventilator-associated pneumonia	1.42 (0.83–2.45)
Age	1.02 (0.99–1.05)
Administration of dexamethasone	1.25 (0.72–2.18)
Administration of tocilizumab	0.71 (0.32–1.60)
New renal replacement therapy	0.99 (0.60–1.63)

Note. CI, confidence interval.

Among the 136 patients, 93 (68.4%) had VAPs. This rate was higher in wave 2, when 28 (71.8%) of 39 patients had VAPs, and in wave 3, when 50 (78.1%) of 64 patients had VAPs, compared with wave 1, when 15 (45.5%) of 33 patients had VAPs. The median time from intubation to first VAP was 10 days (IQR 5–16 days). Of the 137 unique respiratory isolates, methicillin-susceptible *S. aureus* was most common (37.2%, 51 of 137), followed by *Klebsiella* species (21.2%, 29 of 137) and *Pseudomonas aeruginosa* (10.2%, 14 of 137). The full microbiology is shown in Supplementary Table 1. Furthermore, 7 VAPs were complicated by pleural space infections. COVID-19–associated pulmonary aspergillosis was diagnosed in 5 patients. VAPs were not associated with mortality (unadjusted HR, 1.45; 95% CI, 0.85–2.46), including in the multivariate model (adjusted HR, 1.42; 95% CI, 0.83–2.45) (Table 3B).

Furthermore, 125 (91.9%) of the 136 patients received antibiotics while supported with ECMO. Antifungal therapy was administered to 37 patients (27.2%). The mean antibiotic use was 1,031 DOT per 1,000 ECMO days (SD, 496) and the mean antifungal use was 85 DOT per 1,000 ECMO days (SD, 203). Use by specific antimicrobial is shown in Supplementary Table 2 (online). The most commonly used antibiotics were carbapenems (224 DOT per 1,000 ECMO days), parenteral vancomycin (172 DOT per 1,000 ECMO days), first-generation cephalosporins (161 DOT per 1,000 ECMO days), and β-lactam–β-lactamase inhibitor combinations (151 DOT per 1,000 ECMO days).

In our analysis of how BSIs were treated, 18 isolates were excluded for treatment <7 days due to patient death or transfer. The management of the remaining 124 isolates is shown in Table 4. Of the 26 *S. aureus* cases and 13 *Candida* cases, 16 (61.5%) and 7 (53.8%) were treated until decannulation, respectively. Only 13 (40.6%) of 32 cases of non-*Pseudomonas* gram-negative bacilli, 2 (40.0%) of 5 cases of *P. aeruginosa,* 8 (23.5%) of 34 cases of enterococci, and 1 (16.7%) of 6 cases of coagulase-negative staphylococci were treated as such. Relapse occurred in 2 (40.4%) of 5 patients with *P. aeruginosa,* in 2 (33.3%) of 6 patients with coagulase-negative staphylococci, in 4 (30.8%) of 13 patients with *Candida*, in 9 (26.5%) of 34 patients with enterococci, and in 8 (25.0%) of 32 patients with non-*Pseudomonas* gram-negative bacilli. Relapse typically occurred several weeks after initial blood culture positivity (median, 24 days; IQR, 13–36 days). Persistence of infection occurred in 3 (50.0%) of 6 cases with coagulase-negative staphylococci, in 4 (30.8%) of 13 cases with *Candida*, in 9 (28.1%) of 32 cases with non-*Pseudomonas* gram-negative bacilli, and in 6 (17.6%) of 34 patients with enterococci.

**Table 4.** Treatment and Relapses of Bloodstream Infections

Organism	No. of Isolates	Treatment Continued Until Decannulation, No. (%)	Treatment Stopped Before Decannulation, No. (%)	Relapses, No. (%)	Relapses Off Antibiotics, No. (%)	Days to Relapse, Median (IQR)	Persistent Bloodstream Infections, No. (%)
*Staphylococcus aureus*	26	16 (61.5)	10 (38.5)	4 (15.4)	2 (7.7)	30 (20–37)	1 (3.8)
Enterococci	34	8 (23.5)	26 (76.5)	9 (26.5)	7 (20.1)	25 (23–42)	6 (17.6)
Coagulase-negative staphylococci	6	1 (16.7)	5 (83.3)	2 (33.3)	1 (16.7)	23 (22–23)	3 (50.0)
Other gram-positive organisms	7	1 (14.3)	6 (85.7)	0 (0)	0 (0)	N/A	0 (0)
*Pseudomonas aeruginosa*	5	2 (40.0)	3 (60.0)	2 (40.0)	1 (20.0)	18 (13–23)	0 (0)
Other gram-negative bacilli	32	13 (40.6)	19 (59.4)	8 (25.0)	4 (12.5)	21 (15–29)	9 (28.1)
*Candida*	13	7 (53.8)	6 (46.2)	4 (30.8)	2 (15.4)	24 (11–39)	4 (30.8)

Note. IQR, interquartile range.

## Discussion

In this study, BSIs and VAPs were common complications in COVID-19 patients supported with ECMO. The risk of BSIs increased with longer duration of ECMO support, with almost half of patients cannulated for ≥14 days developing a BSI. The overall mortality rate was 55.1%.

Our analyses were focused on BSIs due to concerns with accurately defining VAPs in severe COVID-19 pneumonia.^20^ Previous studies that investigated BSIs in adult ECMO recipients have reported incidence ranging from 2.98 to 18.8 BSIs per 1,000 ECMO days.^13–15,22–24^ We detected a higher incidence of 29.5 BSIs per 1,000 ECMO days. Increased HAI rates, particularly of CLABSIs, have been described during the COVID-19 pandemic.^25,26^ These increased HAI rates have been attributed to deleterious changes in routine infection prevention practices,^25,26^ and CLABSI rates in non-ECMO patients at our center have risen during the pandemic as well. Patient-related factors include immunosuppression from routine use of dexamethasone and interleukin-6 inhibitors for COVID-19. COVID-19 itself may also predispose to BSIs compared to other indications for ECMO. Although coinfections are uncommon in COVID-19 patients, secondary infections during hospitalization are comparatively more frequent.^27,28^

Enterococci, Enterobacterales, and *S. aureus* were the most common BSI pathogens. Previous reports on the microbiology of BSIs in ECMO recipients are limited. In a study of 139 ECMO-supported patients, *Candida* were the most common BSI pathogen, comprising 37.5% of cases.^20^ Another analysis of 220 ECMO recipients reported high rates of *P. aeruginosa,* comprising 21.3% of BSIs.^14^ Similarly, in a study of 267 ECMO recipients, *Candida* and glucose nonfermenting gram-negative bacilli were most frequent, though only 26 BSIs occurred.^24^ In contrast, *Candida* and glucose nonfermenting gram-negative bacilli were relatively uncommon in our cohort, comprising 10.6% and 7.0% of BSI isolates, respectively. These differences may reflect unique hospital-specific microbiology; most published studies were conducted at a single center. A registry study collected microbiologic data across >100 hospitals and reported that *Candida, P. aeruginosa* and *S. aureus* were the most common pathogens causing HAIs in adult ECMO patients.^12^ However, the specific microbiology of BSIs is not known because body-site–specific culture data were not provided. There may also be an inherent impact from COVID-19 infection; in a study of 48,902 hospitalized COVID-19 patients, Enterobacterales, enterococci, and *S. aureus* were the most common causes of hospital-acquired BSIs, similar to our findings.^28^ Varying selection pressures from antimicrobial prophylaxis is also possible.

BSIs and VAPs were not associated with increased mortality. Possible explanations include a critically ill population with high baseline mortality, as well as close clinical monitoring in an ICU with rapid identification of HAIs and initiation of empiric antimicrobial therapy. Our study may also have been underpowered to detect a mortality difference.

BSI relapse was common—it occurred in >20% of cases, typically several weeks after index-positive blood culture. Although catheter exchange or removal is a central tenet of managing BSIs with an indwelling line, this cannot be readily done for patients on ECMO. Colonization of the circuit may therefore occur, which predisposes the patient to BSI persistence and relapse, and colonization has been associated with poorer outcomes.^29–31^ Further interpretation of our results is limited because treatment duration and period of observation for relapse were often constrained by mortality; >90% of patients who died were still being supported with ECMO. The higher relapse rates observed with *P. aeruginosa,* coagulase-negative staphylococci and *Candida* may also be due to small patient numbers. However, the risk of BSI relapse should be recognized, and extended therapy may be considered to prevent this complication, balanced against the adverse effects from prolonged antimicrobial treatment.

Few studies have investigated HAIs in COVID-19 patients supported with ECMO, and none have described their epidemiology and microbiology in detail. Our findings are consistent with the existing literature suggesting that HAIs are common in this population. In a study of 302 patients with COVID-19 who received ECMO, 49% of their patients had a bacteremia and 85% had a VAP.^17^ In a single-center study of COVID-19 ECMO-supported patients, the incidences of BSIs and VAPs were 21.8 and 15.6 per 1,000 ECMO days, respectively.^18^ However, only 17 patients were included in this study. Our study adds further evidence that COVID-19 patients receiving ECMO frequently develop BSIs and VAPs, and it is the first to report their microbiology and to explore their impact on patient outcomes.

We detected a high burden of antibiotic use in our patients, >1,000 DOT per 1,000 ECMO days. Antibiotic use included frequent use of broad-spectrum agents, namely carbapenems, β-lactam–β-lactamase inhibitor combinations and parenteral vancomycin. However, only 2 patients developed *C. difficile* infection. This finding may be related to insufficient follow-up time due to high patient mortality and rapid transfer to other hospitals after ECMO decannulation. Concerns regarding antibiotic overuse in COVID-19 patients have been raised,^32^ and studies have reported that most hospitalized patients are prescribed antibiotics.^28,33^ Due to the frequency of HAIs in COVID-19 patients supported with ECMO, stewardship strategies for judicious antimicrobial use and therapy de-escalation are critical. Shah et al^34^ implemented an antimicrobial prophylaxis protocol for all ECMO patients at their center. Bundled with stakeholder meetings and prospective audit and feedback, the intervention led to decreases in broad-spectrum antimicrobial prophylaxis without increased HAI or death. This intervention was restricted to antimicrobial prophylaxis, which is not supported by guidelines and is not offered at our center. However, similar stewardship strategies using local microbiology data to optimize antimicrobial use should be prioritized, such as guidelines for empiric antimicrobial therapy specific to ECMO-supported patients and routine prospective audit and feedback.

This study had several limitations. We used a single-center design with a relatively small sample size. However, ECMO is a limited resource provided in specialized centers for patients with severe respiratory failure refractory to conventional management. Moreover, we included all COVID-19 patients supported by ECMO through 3 pandemic waves at the largest adult ECMO center in Canada. To our knowledge, it is the largest study to date specifically investigating HAIs in COVID-19 ECMO-supported patients. We did not have a comparator group of critically ill patients who did not receive ECMO; during the height of the pandemic, our ICU was functionally restricted to ECMO patients; therefore, we could not determine the specific impact of ECMO on HAI rates and microbiology. We could not accurately calculate VAP incidence because standard definitions are challenging to apply in patients with severe COVID-19 pneumonia.^20^ Consensus criteria for VAPs in COVID-19 patients would be helpful for future studies. We were unable to adjust for severity of lung injury or critical illness in the multivariate model because this information could not be accurately captured retrospectively. Lastly, most of our patients were transferred from regional centers and rapidly repatriated once decannulated from ECMO. Our mortality rate may have been underestimated because deaths after transfer were not recorded.

In conclusion, BSIs and VAPs are common in patients with COVID-19 supported by ECMO. BSIs increase with longer duration of ECMO cannulation and are frequently associated with relapse. ECMO-capable centers caring for COVID-19 patients should be aware of the risk of BSIs and VAPs, and the substantial associated burden of antimicrobial use in cannulated patients. Prevention and stewardship interventions, guided by local microbiology, are encouraged. Larger multicenter studies with granular patient-level data are needed to further characterize HAIs, to investigate their risk factors, and to determine their optimal treatment.

## Supplementary material

For supplementary material accompanying this paper visit https://doi.org/10.1017/ice.2022.290. click here to view supplementary material
