# Supplementary material for: Bloodstream infection and ventilator-associated pneumonia in patients with coronavirus disease 2019 (COVID-19) supported by extracorporeal membrane oxygenation
Source: Infect Control Hosp Epidemiol. 2022 Dec 1;44(9):1443–50. doi: 10.1017/ice.2022.290 (PMC10507515; doi:10.1017/ice.2022.290)
Supplement: Supplementary file 1 [file S0899823X22002902sup001.docx]

**Supplementary Table 1:** Microbiology of ventilator-associated pneumonias

| **Microorganism (%)** | **Number (n=137)** |
| --- | --- |
| **Gram positive** | **59 (43.1)** |
| Methicillin-susceptible *Staphylococcus aureus* | 51 (37.2) |
| Methicillin-resistant *Staphylococcus aureus* | 4 (2.9) |
| *Streptococcus pneumoniae* | 3 (2.2) |
| Other gram positive organisms | 1 (0.07) |
| **Gram negative** | **77 (56.2)** |
| *Klebsiella* species | 29 (21.2) |
| *Pseudomonas aeruginosa* | 14 (10.2) |
| *Citrobacter species* | 7 (5.1) |
| *Escherichia coli* | 7 (5.1) |
| *Haemophilus influenzae* | 4 (2.9) |
| *Acinetobacter baumannii* | 3 (2.2) |
| *Enterobacter cloacae* | 3 (2.2) |
| Other gram negative organisms | 10 (7.3) |
| ***Candida**** | **1 (0.07)** |
| *Candida albicans* | 1 (0.07) |

*Although *Candida* are generally not considered pathogenic pulmonary organisms, one patient was diagnosed with and treated for *Candida albicans* respiratory infection and empyema

**Supplementary Table 2:** Antimicrobials administered to extracorporeal membrane oxygenation-supported patients

| **Antimicrobial** | **Total days of therapy per 1000 ECMO days** |
| --- | --- |
| **Antibiotics** | **1041** |
| Carbapenems | 224 |
| Vancomycin, parenteral | 172 |
| First-generation cephalosporins | 161 |
| Beta-lactam/beta-lactamase inhibitor combinations | 151 |
| Penicillins | 118 |
| Higher-generation cephalosporins | 103 |
| Fluoroquinolones | 81 |
| Tigecycline | 56 |
| Aminoglycosides | 39 |
| Vancomycin, oral | 22 |
| Trimethoprim-sulfamethoxazole | 12 |
| Doxycycline | 10 |
| Colistin | 7 |
| Linezolid | 2 |
| Macrolides | 1 |
| **Antifungals** | **130** |
| Azoles | 65 |
| Echinocandins | 63 |
| Amphotericin B | 2 |

ECMO = extracorporeal membrane oxygenation
